# Over-the-counter hearing aids and integrated health-monitoring sensors: a review of clinical evidence and implementation

**DOI:** 10.3389/fdgth.2026.1771022

**Published:** 2026-07-29

**Authors:** Naoya Itatani, Melissa Zavaglia

**Affiliations:** Neural Interfacing Group, Munich Institute of Robotics and Machine Intelligence, Technical University of Munich, Munich, Germany

**Keywords:** gait analysis, health monitoring, hearing aid fitting, over-the-counter hearing aids, wearable sensors

## Abstract

The FDA's 2022 over-the-counter (OTC) hearing aid rule created a new access pathway for adults with mild to moderate hearing loss. Hearing aids have also begun incorporating health-monitoring sensors. This PRISMA-guided narrative review evaluates the clinical evidence for OTC hearing aids and the validation status of health sensors in hearing aids, based on 59 studies identified from PubMed, Scopus, Web of Science, and the Cochrane Library and published between 2020 and 2026. In most randomized trials, self-fitted OTC hearing aids produced outcomes noninferior to professionally fitted devices, though one trial found audiologist-fitted hearing aids achieved better results. Consumer adoption has been incremental, with most OTC users still seeking professional support. Usability assessments found that many older adults had difficulty completing self-fitting tasks without error, and current self-fitting interfaces may not guide users toward optimal amplification. Accelerometry-based hearing aid sensors for gait analysis and energy expenditure demonstrated accuracy comparable to established reference and consumer devices. A prototype multi-sensor platform successfully measured temperature, oxygen saturation, heart rate, and respiration rate. Health sensors in hearing aids are technically validated but clinically unproven, as no study has linked sensor data to improved patient outcomes. If outcome studies confirm these uses, sensor-equipped hearing aids could become useful in geriatric medicine, cardiovascular monitoring, and fall prevention, linking audiology to wider medical care. Getting there will require prospective clinical trials, standard validation methods, and regulatory frameworks for dual-purpose devices.

## 1 Introduction

Hearing loss is one of the most common chronic conditions affecting older adults. In the United States, approximately 38 million adults have some degree of hearing loss, but fewer than 20% of those who could benefit from hearing aids use them (1). Globally, the World Health Organization estimates that over 1.5 billion people have some degree of hearing loss, with the greatest shortage of intervention in low- and middle-income countries (2). The reasons for low utilization include high cost, stigma, and a lack of perceived need (3). Untreated hearing loss is also associated with increased risk of cognitive decline and dementia. The 2020 Lancet Commission on dementia identified hearing loss as having the highest population attributable fraction among modifiable risk factors (4), and a meta-analysis found that hearing aid use was associated with a 19% reduction in the long-term hazard of cognitive decline across over 126,000 participants (5). These associations, combined with the scale of the care gap, highlight the urgent need for improving hearing aid access. For decades, hearing aids required a prescription and professional fitting, which placed both financial and logistical barriers between the device and the person who needed it.

In August 2022, the U.S. Food and Drug Administration (FDA) established a new regulatory category for over-the-counter (OTC) hearing aids, allowing adults aged 18 and older with perceived mild to moderate hearing loss to purchase and self-fit hearing aids without a medical evaluation or professional fitting (6). This was the most significant regulatory change in hearing aid access in the United States in decades, and it opened the market to consumer electronics companies alongside traditional hearing aid manufacturers.

At the same time, hearing aid technology has expanded beyond amplification. Modern hearing aids increasingly incorporate health-monitoring sensors, including accelerometers for physical activity and gait tracking and photoplethysmography (PPG) modules for heart rate measurement. Since hearing aids are worn throughout the day, they have the potential to serve as health monitoring platforms without requiring an additional device.

Although OTC access and sensor integration are usually discussed separately, both reflect the same underlying shift. Hearing aids are moving from prescription medical devices fitted by professionals toward consumer devices that people buy, fit, and increasingly rely on for more than hearing. This review focuses on these two developments and the clinical evidence behind them rather than the wider digital health ecosystem, since they are the changes most directly reshaping who uses hearing aids and what those devices are expected to do. Together, they raise important questions about what the evidence supports. Are self-fitted OTC hearing aids effective for their intended users? How accurate are health-monitoring sensors when embedded in hearing aids rather than purpose-built research devices? What challenges arise when these devices move from clinical trials to everyday use?

Despite this shared direction, the clinical evidence behind OTC devices and the validation status of the sensors they carry have not been assessed together. By setting out what the current evidence does and does not support, this review is intended for the wider hearing and aging care community, the developers building these devices, and the researchers and policymakers designing how OTC and sensor-equipped hearing aids are evaluated, while giving prospective users a realistic view of what these devices can do.

This review evaluates the clinical evidence for OTC hearing aids and the validation status of health-monitoring sensors in hearing aids, covering the period from 2020 to 2026. A PRISMA-guided narrative review approach was used to systematically identify and assess the available literature. The review is organized as follows. Section 3 provides the regulatory and access context for OTC hearing aids, including international comparisons. Section 4 presents the clinical evidence from OTC hearing aid trials, with assessment of study design, sample characteristics, and potential bias. Section 5 reviews sensor validation evidence organized by modality. Section 6 examines implementation challenges including professional involvement, usability, consumer perspectives, and data privacy. Section 7 discusses the findings and identifies research priorities, and Section 8 presents the conclusions.

## 2 Methods

This is a narrative review, conducted with a systematic and documented literature search. The search and study selection are reported with reference to the Preferred Reporting Items for Systematic Reviews and Meta-Analyses (PRISMA) 2020 guideline (7), which was used as a reporting framework to keep the search transparent and reproducible.

A narrative approach was used since the review addresses several questions across two distinct areas rather than a single focused question. It examines both the clinical effectiveness of over-the-counter hearing aids and the validation of health-monitoring sensors built into hearing aids. The evidence for these two questions comes from different study designs and outcomes with no shared measure, ranging from randomized and crossover trials of hearing outcomes to accuracy studies of gait, fall, and physiological sensors. The purpose is to bring this evidence together and set out what it does and does not support. No meta-analysis or other statistical pooling was performed, as the included studies report no common outcome that could be combined. A completed PRISMA 2020 checklist is provided in the Supplementary material.

### 2.1 Search strategy

Systematic searches were conducted in PubMed, Scopus, Web of Science, and the Cochrane Library for articles published between January 2020 and May 2026. Searches were performed in May 2026 and applied to title, abstract, and keyword fields across all databases.

Two search domains were defined. For OTC hearing aid evidence, the following terms were combined: (“over-the-counter hearing aid*” OR “OTC hearing aid*” OR “self-fitting hearing aid*” OR “direct-to-consumer hearing aid*”) AND (clinical trial OR effectiveness OR outcome* OR noninferiority OR satisfaction OR comparison). For health sensor validation in hearing devices, terms included: (“hearing aid” OR “hearable*” OR “ear-worn” OR “in-ear”) AND (“wearable sensor*” OR “fall detection” OR “gait” OR “acceleromet*” OR “photoplethysmogra*” OR “PPG” OR “heart rate” OR “activity monitor*”) AND (validation OR accuracy OR sensitivity OR specificity OR feasibility).

In addition to database searching, reference lists of all included studies were screened for relevant sources not captured by the primary search. Authoritative regulatory and governmental documents (e.g., FDA Final Rule, NASEM reports) were identified through purposive searching and included without date restriction where directly relevant to OTC hearing aid policy. These sources were not subject to the database screening process and are reported separately in the PRISMA flow diagram.

### 2.2 Eligibility criteria

Studies were included if they: (a) were peer-reviewed original research, systematic review, or meta-analysis; (b) reported clinical outcomes for OTC or self-fitting hearing aids, or reported validation data for health-monitoring sensors in hearing aids; and (c) were published in English. For sensor validation studies, the device under investigation must be a hearing aid. Studies of generic earables, earbuds, or in-ear prototypes without hearing aid context were excluded. Sensor studies using prescription hearing aids were included, as sensor capabilities validated in prescription devices are expected to transfer to the OTC market.

Studies were excluded if they: (a) were conference abstracts without full-text publication; (b) were manufacturer whitepapers, trade publications, or market research reports; (c) reported only on prescription hearing aids without OTC or self-fitting comparison (except sensor validation studies as noted above); (d) were opinion pieces without original data; or (e) were generic earable sensor studies without hearing aid context.

### 2.3 Study selection and data extraction

**Figure 1:**
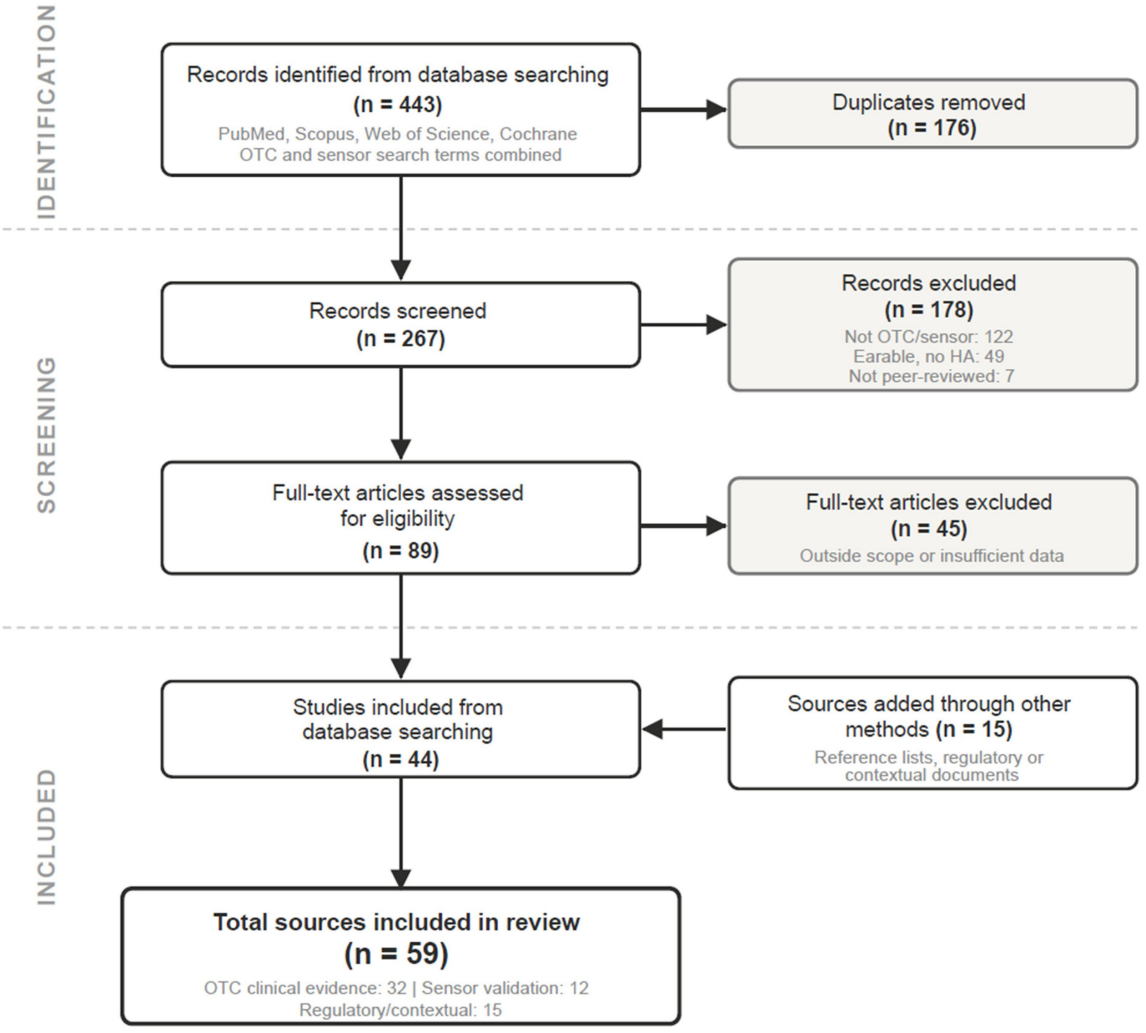
PRISMA flow diagram of study selection. A total of 443 records were identified from database searching (PubMed, Scopus, Web of Science, and Cochrane). An additional 15 sources were added through other methods, comprising reference list screening and regulatory or contextual documents. A total of 59 studies were included in the review, covering OTC clinical evidence and implementation (*n* = 32), sensor validation and health monitoring (*n* = 12), and regulatory and contextual references (*n* = 15).

Results from all database searches were exported into the Zotero reference management system, and duplicate records were identified and removed. Titles and abstracts of all unique records were screened against the eligibility criteria. Full texts of potentially eligible articles were retrieved and assessed against inclusion criteria. For each included study, the following data were extracted: study design, sample size, population characteristics, intervention or device tested, primary outcomes, key findings, and potential sources of bias. The study selection process is summarized in a PRISMA flow diagram (Figure 1). Database searches identified 443 records. After removing 176 duplicates, 267 records were screened by title and abstract, of which 89 were assessed at full text. A total of 44 studies met the inclusion criteria through database searching. An additional 15 sources were added through other methods, comprising reference list screening and regulatory or contextual documents. These sources were assessed against the same eligibility criteria, giving 59 studies included in the review.

### 2.4 Evidence assessment

For OTC clinical trials, study design quality, population representativeness, effect magnitude and precision, and potential sources of bias including industry sponsorship and geographic context were assessed. For sensor validation studies, measurement accuracy, sample characteristics, testing conditions (laboratory vs. real-world), and the gap between technical performance and clinical utility were evaluated.

### 2.5 Risk of bias assessment

Risk of bias was assessed with tools matched to each study's design. Comparative clinical trials were assessed with the Cochrane RoB 2 tool, applying the parallel-group and crossover versions as appropriate and rating the effect of assignment to intervention (8). Sensor validation studies, in which a device measurement was compared against a reference standard, were assessed with QUADAS-2 (9). Descriptive surveys, cross-sectional evaluations, single-arm reports, and bench electroacoustic measurements were assessed narratively rather than with a per-study tool, as these designs either lack a comparator or do not test a device against a reference standard. Each comparative trial was rated across the five RoB 2 domains, and each validation study across the four QUADAS-2 domains together with applicability. No study was excluded on the basis of its rating, and the ratings inform interpretation only.

## 3 OTC regulatory and access context

The primary barrier to hearing aid adoption has been cost. A recent policy analysis reported an average bundled price (the device together with professional services) of about $2,500 for hearing aids and estimated that this places them out of financial reach for roughly 77% of Americans with hearing loss (10). Traditional Medicare does not cover hearing aids, so this cost is largely paid directly by consumers.

The regulatory path to OTC hearing aids began with the 2016 National Academies of Sciences, Engineering, and Medicine (NASEM) report, which identified bundled pricing, limited distribution channels, and a lack of over-the-counter options as key barriers to hearing aid access (11). Following the Over-the-Counter Hearing Aid Act of 2017, the FDA published its final rule in August 2022, allowing adults aged 18 and older with perceived mild to moderate hearing loss to purchase hearing aids directly without a medical examination, audiometric testing, or professional fitting (6). The rule established technical requirements including a maximum output of 111 dB SPL (117 dB SPL with input compression), latency under 15 milliseconds, and user-accessible volume controls. The rule also defined contraindications that must be communicated to consumers, including sudden hearing loss within the previous 90 days, unilateral hearing loss, visible ear abnormalities, ear pain, or drainage (6).

In September 2024, the FDA granted *de novo* authorization for the Apple AirPods Pro 2 to function as an OTC hearing aid device (12). This was the first time a consumer electronics product designed primarily for audio entertainment received regulatory clearance as a hearing aid, expanding the OTC category beyond purpose-built hearing devices. An independent evaluation of the Apple hearing test feature found clinically acceptable accuracy, with 86.5% of thresholds within 10 dB of standard audiometry and reliable test-retest performance in 25 adults with mild-to-moderate hearing loss (13). The test was also significantly faster than standard audiometry (median 5.5 vs. 10 min).

International approaches to hearing aid access differ from the US model in regulation, funding, and delivery. In terms of regulation, the European Union classifies hearing aids as Class IIa medical devices under the Medical Device Regulation, requiring conformity assessment and maintaining professional involvement in the fitting process (14). In terms of funding, the United Kingdom's National Health Service reimburses nearly all hearing aid costs, with assessment and fitting delivered through publicly funded audiology services (15). In Australia, access depends on eligibility for public programs, with individuals not covered bearing the full cost of hearing services and devices (16). South Africa has tested an alternative delivery model, with a randomized trial of direct-to-consumer fitting showing outcomes comparable to traditional fitting (17). These examples illustrate different approaches to balancing consumer access with professional oversight across health systems. The US OTC decision may also influence international markets. Willink et al. (16) predicted that the introduction of OTC hearing aids in the US would drive change in Australia's hearing system, and similar effects may be expected in other countries where consumer access to hearing aids remains limited.

While the FDA rule broadened access, it also placed responsibility on consumers to determine whether OTC hearing aids are appropriate for them. The contraindications listed on device packaging may not reach consumers during online research or at the point of retail purchase. The 2024 clinical practice guidelines from the American Academy of Otolaryngology-Head and Neck Surgery Foundation (AAO-HNSF) recommend that patients with significant asymmetric hearing loss, conductive or mixed hearing loss, or poor word recognition should be evaluated by a specialist rather than using OTC devices (18). McManus et al. (19) found that approximately 10% of US adults have excessive or impacted cerumen, a condition that could affect hearing aid performance and that consumers may not recognize without a professional ear examination. These findings highlight a tension in the OTC model between improving access and ensuring that consumers who need medical evaluation are not overlooked.

## 4 OTC clinical evidence

The FDA's 2022 OTC hearing aid rule created two distinct product categories. Self-fitting devices allow users to adjust amplification through a smartphone application, while preprogrammed devices use manufacturer-set amplification profiles. Since implementation, clinical trials have examined whether these devices deliver meaningful hearing benefit. The evidence below is presented by study design, with attention to limitations that affect how the results can be used in practice. Table 1 summarizes the studies reviewed in this section.

**Table 1:** Summary of OTC clinical evidence.

Study	Design	*n*	Country	Intervention	Primary Outcome	Key Finding	Notes
4.1 Self-fitting versus professionally fitted hearing aids							
Humes et al. (20)	Multisite noninferiority RCT	584	US	Self-fit (2 methods) vs. audiologist best-practice; same HAs all groups	PHAB, HHIE	Both self-fit methods noninferior at 6 weeks and 6 months	Same HAs, not commercial OTC
De Sousa et al. (17)	RCT	64	South Africa	Self-fitting OTC (Lexie Lumen/Bose) vs. audiologist-fit	APHAB, IOI-HA, speech-in-noise	Self-fit noninferior at 6 weeks	Single device, single center
De Sousa et al. (21)	RCT follow-up	44	South Africa	8-month follow-up of De Sousa (17)	APHAB, IOI-HA, speech-in-noise	Sustained noninferiority at 8 months	Same device/center as 2023
Knoetze et al. (22)	Crossover trial	28	South Africa	Self-adjustment vs. *in situ* audiometry; OTC devices	APHAB, IOI-HA, speech-in-noise, REM	Both strategies equivalent; self-adjustment had higher satisfaction	Short-term assessments
Zheng et al. (23)	RCT	64	US	Self-fit vs. professional-fit	REAG, QuickSIN, APHAB, SSQ12	Professional-fit higher REAG at 2 kHz; no functional outcome differences	—
Baltzell et al. (24)	Within-subjects crossover	21	US	Commercial self-fitting OTC vs. audiologist-fit	Self-reported benefit, speech-in-noise	Self-fit noninferior	Industry (Concha Labs)
Wu et al. (25)	RCT	245	US	3 service models×2 technology levels	GHABP	Audiologist-fit significantly better (0.33, 0.32 pts; 95% CI: 0.14–0.52, 0.13–0.51); OTC still positive	Simulated OTC condition
4.2 Fitting approaches and gain characteristics							
Sabin et al. (26)	Field trial (1 month)	75	US	Self-fitting interface (two adjustment wheels)	Gain match, benefit, speech-in-noise	Gain within 1.8 dB overall and 5.6 dB/band of audiologist; no benefit differences	Industry (Bose)
Venkitakrishnan et al. (27)	Randomized crossover	37	US	Evidence-based presets vs. NAL-NL2 vs. PSAP	Listening effort, sound quality, speech recognition	Presets comparable to NAL-NL2; outperformed PSAP; 54% preferred presets	—
Knoetze et al. (28)	Gain evaluation	43	South Africa	6 FDA-approved self-fitting OTC devices	Real-ear gain vs. NAL-NL2 targets	Met 10 dB but not 5 dB criterion; largest deviations at high frequencies; gain stable	No clinical outcomes\*
Pinsonnault-Skvarenina et al. (29)	Electroacoustic evaluation	11 devices	—	11 direct-to-consumer hearing devices	ANSI/CTA-2051 compliance	Most met standard; transparency-attenuation trade-offs varied	Bench test only\*
4.3 Real-world outcomes and broader health effects							
Swanepoel et al. (30)	Cross-sectional survey	656	US	OTC vs. HCP service delivery	IOI-HA	No significant overall difference; HCP longer daily use	Online survey
Picou (55)	Survey (MarkeTrak 2025)	869	US	OTC vs. traditional HA owners	Satisfaction	Traditional 83%; OTC 76%; OTC less satisfied with size/reliability, more with price	—
Sheng et al. (32)	Survey + prospective	255 + 29	US	Self-fitting OTC (Eargo)	SADL	Within range for prescription HAs	Industry (Eargo); survey bias
Mothemela et al. (33)	Community-based model	25	South Africa	Bilateral preset OTC fitted by CHWs	IOI-HA	Above average (use 3.91, benefit 4.46, satisfaction 4.58); 92% extremely helpful	Small sample, no comparison
Jiang et al. (35)	Parallel RCT, open-label	221	China	CHW-delivered OTC HAs vs. waitlist	GDS-15 (depression)	Between-group difference 1.57 (95% CI: 1.41–1.72, *p* < 0.001); improvements in communication, isolation, loneliness	Open-label; waitlist control; HL range beyond OTC indication
Kruger et al. (34)	Cross-sectional evaluation	25	South Africa	Apple AirPods Pro 2 OTC HA Feature	mHealth usability, QuickSIN, REM	High usability (6.7/7); nonsignificant speech-in-noise benefit; gain below NAL-NL2 targets	Digitally literate users only
4.4 Limitations of the current evidence base							
Szatkowski and Souza (36)	Within-subject	30	US	Preprogrammed OTC; aided vs. unaided	DiapixUK, auditory recall, sentence recognition	No improvement in conversation efficiency; recognition near ceiling unaided, lower with default program	No prescription HA comparison

* Study without clinical outcome measures, included for completeness.

APHAB, abbreviated profile of hearing aid benefit; CHW, community health worker; DiapixUK, spot-the-difference conversation task; GHABP, glasgow hearing aid benefit profile; GDS-15, geriatric depression scale (15-item); HA, hearing aid; HCP, hearing care professional; HHIE, hearing handicap inventory for the elderly; HL, hearing loss; IOI-HA, international outcome inventory for hearing aids; OTC, over-the-counter; PHAB, profile of hearing aid benefit; PSAP, personal sound amplification product; QuickSIN, quick speech in noise test; RCT, randomized controlled trial; REAG, real-ear aided gain; REM, real-ear measurement; SADL, satisfaction with amplification in daily life; SSQ12, speech, spatial and qualities of hearing scale (12-item).

### 4.1 Self-fitting vs. professionally fitted hearing aids

The strongest evidence for OTC hearing aids comes from randomized trials comparing self-fitted to audiologist-fitted interventions. Humes et al. (20) conducted the largest study in this area, a multisite noninferiority trial at four US sites randomizing 584 adults aged 50 to 79 with perceived hearing difficulty to one of three groups: audiologist-fit using best practices, or one of two self-fit methods. Participants were stratified by audiometric hearing loss category (normal, mild, or moderate), and all groups received the same hearing aids. At both 6 weeks and 6 months, both self-fit methods yielded outcomes that were noninferior to the professional-fit method on self-reported benefit (Profile of Hearing Aid Benefit) and hearing handicap measures. Clinically meaningful benefit was observed regardless of fitting method.

De Sousa et al. (17) conducted a randomized clinical trial in South Africa comparing a self-fitting OTC hearing aid (Lexie Lumen by Lexie Hearing) to the same device fitted by an audiologist. At six weeks, the self-fitted intervention was noninferior to the audiologist-fitted intervention on self-reported benefit measures [Abbreviated Profile of Hearing Aid Benefit (APHAB) and International Outcome Inventory for Hearing Aids (IOI-HA)] and speech-in-noise performance. A follow-up study by the same group (21) extended these findings to eight months, reporting sustained noninferiority on the same outcome measures. These two trials provide strong evidence, but both used the same device, were conducted at a single center in South Africa, and the same research group conducted both studies.

Knoetze et al. (22) compared two self-fitting strategies in a crossover clinical trial of 28 adults using FDA-approved OTC devices. One strategy let users adjust gain and tone themselves, while the other fit the devices automatically from an in-app hearing test. The two approaches produced similar outcomes, although users who adjusted the devices themselves reported higher satisfaction and longer daily use. The crossover design removes differences between participants, but the study was limited to short-term laboratory and field assessments.

Zheng et al. (23) randomized 71 adults with mild-to-moderate sensorineural hearing loss to self-fit or professional-fit groups, of whom 64 completed the study. The professional-fit group showed significantly higher real-ear aided gain at 2,000 Hz across all input levels, particularly among older participants and those with greater hearing loss severity. However, no significant differences were found on functional outcome measures including the Quick Speech in Noise test (QuickSIN), the APHAB, or the Speech, Spatial, and Qualities of Hearing Scale (SSQ12). This pattern of better acoustic output with professional fitting but similar self-reported benefit is consistent across several OTC trials. It raises a question about whether acoustic match to prescriptive targets is the right measure of OTC success.

Baltzell et al. (24) used a within-subjects crossover design to compare a commercially available self-fitting OTC hearing aid against an audiologist-fitted intervention. Self-fitted outcomes were noninferior to audiologist-fitted outcomes for both self-reported benefit and speech-in-noise performance. The authors noted that some deviations from best audiological practices in their clinician-fitted condition may have influenced results, which makes the size of the self-fitting advantage harder to judge.

Wu et al. (25) conducted a randomized trial at two US sites in which 245 adults with mild-to-moderate hearing loss completed the study. Participants were assigned to one of three service models (full audiologist fitting, limited audiologist support for OTC devices, or independent OTC use) and two technology levels (high-end and low-end). Unlike the noninferiority findings from the trials above, the audiologist-fitted group scored significantly higher on the Glasgow Hearing Aid Benefit Profile (GHABP) than both OTC groups (by 0.33 and 0.32 points on a 5-point scale, 95% CI: 0.14 to 0.52 and 0.13 to 0.51, respectively). The two OTC groups did not differ significantly from each other. However, OTC scores were still close to 4 out of 5, indicating positive outcomes even without full audiologist involvement. High-end and low-end hearing aids yielded similar outcomes regardless of service model. One limitation is that the OTC condition was simulated using prescription hearing aids rather than commercially available OTC devices, which may have affected how participants experienced the self-fitting process.

### 4.2 Fitting approaches and gain characteristics

The self-fitting approach used in several OTC trials was first validated by Sabin et al. (26), who tested an interface where users select their own signal processing parameters through two adjustment wheels. Over a one-month field trial, participants in the self-fitting group selected gain within 1.8 dB overall and 5.6 dB per frequency band of audiologist-selected parameters, with no differences in clinical measures of benefit or speech perception in noise. This study established that users can select effective amplification without professional assistance.

Venkitakrishnan et al. (27) compared evidence-based presets developed for OTC hearing aids against a conventional NAL-NL2 prescription fitting and a personal sound amplification product (PSAP) in 37 participants using a randomized crossover design. The evidence-based presets performed comparably to NAL-NL2 across most outcome measures and outperformed the PSAP on listening effort, sound quality, and real-world speech recognition. Most participants (54%) preferred the evidence-based presets over the PSAP.

Knoetze et al. (28) examined gain characteristics of six FDA-approved self-fitting OTC hearing aids in 43 participants. Gain met the 10 dB SPL tolerance criterion from NAL-NL2 targets but not the stricter 5 dB SPL criterion, with the largest deviations at higher frequencies. Gain remained stable over time, indicating that users make limited adjustments after initial setup. This suggests that fitting algorithms allowing gradual adjustment over time may be needed, as users tend not to increase amplification on their own.

At the device level, Pinsonnault-Skvarenina et al. (29) evaluated the electroacoustic and acoustic transparency performance of 11 direct-to-consumer hearing devices. Most devices met the requirements of the ANSI/CTA-2051 standard, but trade-offs between transparency and attenuation features varied across devices, suggesting that device selection should be guided by individual hearing profiles and needs.

### 4.3 Real-world outcomes and broader health effects

Swanepoel et al. (30) compared self-reported outcomes between OTC and conventional hearing care professional (HCP) clients in a cross-sectional survey of 656 hearing aid users. After controlling for demographic and hearing variables, no significant difference in overall outcomes was found between the two groups on the IOI-HA, although HCP clients reported longer daily use.

Several studies assessed OTC outcomes in everyday use and at population level. Picou (31) analyzed benefit and satisfaction data from the MarkeTrak 2025 survey, comparing OTC and traditional hearing aid owners. Traditional hearing aids showed stable satisfaction rates at 83%, while OTC hearing aids had slightly lower satisfaction at 76%. OTC users were less satisfied with device size and reliability but more satisfied with price.

Sheng et al. (32) reported on real-world satisfaction with a self-fitting OTC hearing aid in two investigations, a survey of 255 active users and a prospective study of 29 new users followed for at least 10 weeks. Satisfaction scores on the Satisfaction with Amplification in Daily Living (SADL) scale fell within the range previously reported for prescription hearing aids. However, the survey component recruited existing satisfied users, which biases the results toward positive outcomes, and all participants used the same device (Eargo-powered), limiting how broadly the findings apply. It should also be noted that all authors were affiliated with Eargo.

Mothemela et al. (33) tested a community-based OTC hearing aid fitting model in Khayelitsha, South Africa, where community health workers fitted 25 participants with bilateral preset OTC devices. IOI-HA scores were above the standardized average (daily use: 3.91; benefit: 4.46; satisfaction: 4.58), with 92% reporting the devices as extremely helpful. This study is one of the few to assess OTC hearing aids in a low-income, non-Western setting, a group that is rarely included in OTC clinical studies. The small sample size and lack of a comparison group, however, make the conclusions less certain.

Kruger et al. (34) provided the first independent clinical evaluation of the Apple AirPods Pro 2 as an OTC hearing aid in 25 adults with mild-to-moderate hearing loss. Usability was high (mean mHealth App Usability Questionnaire score 6.7 out of 7), but speech-in-noise benefit was not significant and real-ear gain fell below prescriptive targets. These findings suggest that consumer electronics hearing aids, while accessible and well-received by users, may face different performance challenges than purpose-built OTC devices.

Beyond hearing-specific outcomes, one trial examined the effect of OTC hearing aid on depression. Jiang et al. (35) conducted a parallel, randomized, controlled, open-label trial in China, randomizing 221 older adults (≥60 years) with mild-to-moderately-severe hearing loss to receive community health worker-delivered OTC hearing aids (*n* = 109) or a waitlist control (*n* = 112). At six months, the intervention group showed significant improvement in depression symptoms measured by the Geriatric Depression Scale (GDS-15), with a between-group difference of 1.57 points favoring the intervention (95% CI: 1.41 to 1.72, *p* < 0.001), with similar improvements in communication function, social isolation, and loneliness. Approximately 80% maintained more than four hours of daily use, and no adverse events were reported. This is the first randomized trial to link OTC hearing aids to improvement in depression symptoms. However, several design features limit the conclusions. The open-label design means both participants and assessors knew who received hearing aids. The waitlist control does not account for placebo effects of wearing a device. The study population (rural China) may not generalize to other settings. The hearing loss range also extended to moderately severe, which is beyond the FDA's OTC indication for mild-to-moderate loss.

### 4.4 Limitations of the current evidence base

OTC clinical trials share several common limitations. First, there is geographic concentration. The largest and best-designed trials were conducted in South Africa (17, 21, 22, 28, 33), and the depression trial was conducted in China (35). US-based evidence has grown with the publication of the Humes et al. (20) and Wu et al. (25) trials, but remains limited overall. Second, most sample sizes are small [21 to 75 participants in most controlled trials, with the Humes (*n* = 584), Wu (*n* = 245), and Jiang (*n* = 221) trials being exceptions], which makes it difficult to test whether results differ for specific groups of patients. Third, few studies include follow-up beyond six months, leaving questions about sustained benefit, device abandonment, and hearing progression unanswered. Fourth, most trials used a single device brand, making it difficult to generalize findings across the growing OTC market. Additionally, many self-fitting trials relied on app-based interfaces, which assume a level of digital comfort that may not generalize to all older adults. Szatkowski and Souza (36) evaluated preprogrammed OTC hearing aids, which do not involve self-fitting, in 30 adults using a conversation efficiency task and speech recognition measures. No significant improvement was found with OTC use compared to the unaided condition, and sentence recognition scores were near ceiling unaided but lower with the default hearing aid program. This suggests that the positive findings from self-fitting trials may not extend to all OTC device types.

Existing trials mostly included older adults with mild-to-moderate sensorineural hearing loss, with few younger adults, non-English speakers, people with cognitive impairment, or those with complex audiological profiles. A 2018 review of direct-to-consumer hearing device outcomes found consistently positive results in clinical and self-reported measures among older adults, although the evidence was largely short-term and of low quality (37). The post-2022 evidence reviewed here has improved in study design, but many of the same limitations persist. Several registered clinical trials identified during our literature search have not yet reported results, including comparisons of delivery models (NCT05527730, NCT04671381) and evaluations of consumer electronics hearing aids (NCT07044518), suggesting that the evidence base will expand in the coming years.

## 5 Sensor validation in hearing aids

Beyond amplification and connectivity, modern hearing aids increasingly include health-monitoring sensors, particularly accelerometers and, in some devices, photoplethysmography (PPG) sensors for heart rate monitoring. Hearing aid accelerometers were originally incorporated for automatic environmental classification and gain adjustment, allowing devices to optimize settings based on whether the user is stationary or in motion (38). More recently, these sensors have been investigated for health monitoring applications. The ear canal provides reliable skin contact and proximity to major blood vessels, and the temporal bone transmits body accelerations effectively. Because users already wear hearing aids throughout the day, health monitoring can be added without requiring a separate wearable. This section reviews the validation evidence for each sensor modality in hearing aids. Table 2 summarizes the included sensor validation studies.

**Table 2:** Sensor validation summary in hearing aids.

Sensor Type	Study	Device	Device specifications	*n*	Accuracy Metric	Setting	Clinical Readiness
Gait analysis (temporal parameters)	Seifer et al. (39)	Hearing aid accelerometer (EarGait toolbox)	WS Audiology HA, integrated IMU; triaxial accelerometer ±16 g, gyroscope ±2,000°/s, 200 Hz (equivalent results at 50 Hz)	48	Mean absolute stride time error 12 ± 32 ms vs. optical motion capture; 99.8% sensitivity for initial contact detection	Laboratory	Validated
Gait analysis (step length, gait speed)	Seifer et al. (40)	Hearing aid accelerometer (ML models)	Same HA and dataset as Seifer et al. (39); triaxial accelerometer, 200 Hz; robust across sampling rates	48 (27 young, 21 older)	Mean absolute step length error 3.9 cm; gait speed error 5.4% (±4.0%) on walking bout level	Laboratory	Validated; sensitive to walking speed
Gait (effect of amplification on gait)	Seifer et al. (41)	Hearing aid	Not reported	25	No significant differences in gait velocity or step length between aided and unaided conditions	Controlled	Negative finding: amplification does not affect gait parameters
Pedometry	Sloan et al. (42)	Hearing aid accelerometer vs. ActiGraph	WSA Signia Pure 312 HA; manufacturer pedometer algorithm; triaxial accelerometer (bilateral); sampling rate not stated	4	Left-right HA step counts consistent with each other; actual counts varied above and below ActiGraph values	Real-world (14 days; 10–14 h/day wear)	Needs improvement; step count algorithm inaccurate
Clinical mobility tests	Sloan et al. (43)	Hearing aid accelerometer	Same HA as Sloan et al. (42); triaxial accelerometer, 8 Hz; vendor-provided processing	13 (1 lab, 12 community)	Matched stopwatch-based clinical measures within MCID for Five Times Sit-to-Stand, Timed Up and Go, and 2-minute walk	Laboratory + community	Validated for standard clinical mobility assessments
Fall detection	Burwinkel et al. (44)	Starkey Livio AI (inertial sensors)	Starkey Livio AI i2400 BTE 13; integrated IMU (accelerometer + gyroscope), 104 Hz; two firmware variants (A, B) evaluated by offline emulation	10 (young adults, mean age 23.8)	Sensitivity 92.1% (variant 1) and 80.0% (variant 2); low false alarm rates during ADLs	Laboratory (simulated falls + ADLs)	Laboratory only; no real-world fall detection data published
Energy expenditure	Stutz et al. (45)	Sonova hearing aid accelerometer	Audéo Fit (Sonova AG); triaxial accelerometer, 200 Hz	60 (middle-aged to older adults)	Comparable to hip-worn research device when activity type known; mean bias +61 kcal over 10 h wearing day (LoA −241 to +363 kcal, MAE 131 kcal); comparable to wrist-worn Fitbit	Laboratory (13 ADLs)	Validated; accuracy varies by activity type
Multi-sensor (temperature, SpO2, heart rate, respiration rate)	Schroeder et al. (46)	Prototype receiver-in-canal hearing aid	Prototype in Starkey RIC HA; TMP117M temperature sensor; MAXM86161 PPG with MAX32664 hub (heart rate, SpO2); LIS2DS12 accelerometer; existing LSM6DSO IMU (respiration); PPG 100 Hz, readings every 15 min	HR: 29, SpO2: 12, respiration: 17, comfort: 14	All modalities met accuracy standards set by regulatory bodies or research community; well tolerated for multi-hour wear; no additional battery charging needed	Controlled (single prototype)	Proof of concept; no independent replication

ADL, activities of daily living; BTE, behind-the-ear; HA, hearing aid; HR, heart rate; IMU, inertial measurement unit; LoA, limits of agreement; MAE, mean absolute error; MCID, minimal clinically important difference; ML, machine learning; PPG, photoplethysmography; RIC, receiver-in-canal; SpO2, oxygen saturation. In the accuracy column, sensitivity denotes detection sensitivity, the proportion of true events correctly identified, and is distinct from the acoustic or sensor sensitivity of the hearing aid hardware. Device specifications are listed only where the source study reported them. Clinical readiness reflects the study's own findings and limitations as reported. No study has linked hearing aid sensor data to improved clinical outcomes.

### 5.1 Accelerometry: gait, fall detection, and activity monitoring

Accelerometry is the most studied sensor modality in hearing aids, with applications in gait analysis, fall detection, and activity monitoring. The ear-worn position offers an advantage over wrist-worn devices since head motion during walking is more stable than arm swing, which changes when carrying objects or putting hands in pockets.

For gait analysis, the most detailed work was reported by Seifer and colleagues. Seifer et al. (39) developed EarGait, an open-source toolbox for estimating temporal gait parameters from hearing aid accelerometers. In 48 participants, the system achieved a mean absolute stride time error of 12 ± 32 milliseconds compared to optical motion capture, with 99.8% sensitivity for detecting initial contact events. A follow-up study (40) extended this work to step length and gait speed estimation using machine learning models, achieving a mean absolute step length error of 3.9 cm and gait speed error of 5.4% (± 4.0%) on a walking bout level. The model performed well across different age groups and sampling rates but was sensitive to walking speed. Most recently, Seifer et al. (41) tested whether hearing aid amplification itself affects gait, finding no significant differences in gait velocity or step length between aided and unaided conditions in controlled settings.

Sloan and colleagues assessed hearing aid accelerometers for clinical mobility tests. Sloan et al. (42) compared pedometry from hearing aid accelerometers to an ActiGraph device over 14 days in older adults. Participants wore hearing aids 10 to 14 h per day, showing good compliance, and the left-right hearing aid step counts were consistent with each other. However, the actual step counts varied above and below the ActiGraph values, suggesting the hearing aid algorithm needs further improvement. In a follow-up feasibility study, Sloan et al. (43) tested hearing aid accelerometers on three clinical mobility assessments (Five Times Sit-to-Stand, Timed Up and Go, and 2 min walk). In a single laboratory participant, the algorithm matched stopwatch timing within the minimal clinically important difference for the sit-to-stand and Timed Up and Go tests, and 2 min walk step counts were within about 1.3% of an observer count. The accuracy comparison was limited to this one participant. The 28 older adults' sit-to-stand data were compared only to age and sex normative ranges, with no stopwatch or observer reference, and usable data were available for 12 of them.

For fall detection, Burwinkel et al. (44) tested inertial sensors embedded in Starkey Livio AI hearing aids during simulated falls and activities of daily living in 10 young adults (mean age 23.8 years). One sensor variant achieved 92.1% sensitivity for detecting falls, while a second variant achieved 80.0%. Both maintained low false alarm rates during daily activities. All testing was conducted in the laboratory, and no real-world fall detection data has been published.

Stutz et al. (45) assessed energy expenditure estimation using a Sonova hearing aid accelerometer in 60 middle-aged to older adults performing 13 activities of daily living. When activity type was known, the hearing aid sensor performed comparably to a hip-worn research device. Accuracy varied across different activities, but when averaged over a typical 10-hour wearing day, the hearing aid sensor showed a mean bias of +61 kcal compared to the reference (limits of agreement: −241 to +363 kcal, MAE 131 kcal). This was comparable to a wrist-worn Fitbit, while a wrist-worn Garmin showed similar accuracy but lower precision (limits of agreement: −716 to +989 kcal).

### 5.2 Multi-sensor platforms and readiness assessment

Schroeder et al. (46) built a multi-sensor health monitoring system into an existing receiver-in-canal hearing aid. The system measured four vital signs (body temperature, oxygen saturation, heart rate, and respiration rate). All sensor modalities met accuracy standards set by regulatory bodies or the research community. Wearing the in-canal device for several hours was well tolerated, and because readings were taken only at intervals, additional battery charging was not required throughout the day. This is the only published study to validate heart rate and other vital sign measurements from a hearing aid sensor. However, it was a single prototype tested under controlled conditions, and no independent replication has been reported.

**Figure 2:**
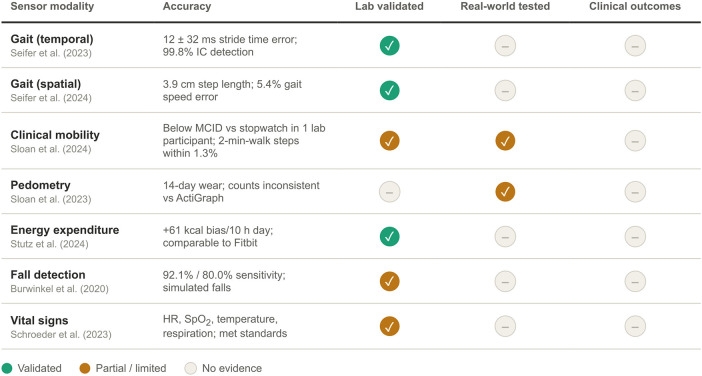
Sensor readiness assessment for health-monitoring modalities integrated into hearing aids. Green circles indicate modalities that met accuracy targets in laboratory or controlled testing. Brown circles indicate partial or limited validation. Empty circles indicate no published evidence. No sensor modality has demonstrated that hearing aid sensor data leads to improved clinical outcomes such as fewer falls, earlier detection of health decline, or better chronic disease management. IC, initial contact; MCID, minimal clinically important difference; HR, heart rate; SpO2, blood oxygen saturation.

Across all modalities, a common pattern is observed. Sensor accuracy has been demonstrated in laboratory settings, and in some cases during short-term real-world use. However, no study has yet shown that continuous health monitoring through hearing aids leads to improved clinical outcomes such as fewer falls, earlier detection of health decline, or better management of chronic conditions. The gap between technical validation and clinical benefit remains the main barrier to treating hearing aid sensors as medical-grade health monitoring tools. Figure 2 summarizes the current readiness status of each sensor modality.

Formal risk of bias assessment reflected these patterns. Across the nine comparative trials, every trial carried at least some concerns, driven mainly by self-reported primary outcomes in studies where the fitting method cannot be blinded, which is inherent to the topic rather than a weakness of the studies (Table 3). Three trials were rated at high risk of bias, De Sousa et al. (21) for differential attrition at long-term follow-up, Knoetze et al. (22) for unconcealed allocation and a mid-study replacement of participants that broke the randomization, and Jiang et al. (35) for an unblinded self-reported depression outcome measured against a waitlist. Among the seven sensor validation studies, three were at low risk of bias against strong reference standards while four were at high risk, and applicability concerns were high for all seven, as validation was conducted in healthy volunteers rather than the older or mobility-impaired populations these devices would monitor (Table 4).

**Table 3:** Risk of bias assessment of comparative clinical trials (cochrane RoB 2).

Study	D1 Randomization	D2 Deviations	D3 Missing data	D4 Outcome measurement	D5 Reported result	Overall
Humes et al. (20)	Low	Some concerns	Some concerns	Some concerns	Low	Some concerns
De Sousa et al. (17)	Some concerns	Some concerns	Low	Some concerns	Low	Some concerns
De Sousa et al. (21)	Some concerns	Some concerns	High	Some concerns	Some concerns	High
Knoetze et al. (22)	High	Some concerns	Low	Some concerns	Low	High
Zheng et al. (23)	Some concerns	Some concerns	Low	Some concerns	Some concerns	Some concerns
Baltzell et al. (24)	Some concerns	Some concerns	Low	Some concerns	Some concerns	Some concerns
Wu et al. (25)	Low	Some concerns	Some concerns	Some concerns	Low	Some concerns
Venkitakrishnan et al. (27)	Some concerns	Some concerns	Low	Some concerns	Some concerns	Some concerns
Jiang et al. (35)	Low	Some concerns	Low	High	Low	High

D1, randomization process; D2, deviations from intended interventions; D3, missing outcome data; D4, measurement of the outcome; D5, selection of the reported result.

**Table 4:** Risk of bias and applicability assessment of sensor validation studies (QUADAS-2).

Study	Patient selection	Index test	Reference standard	Flow and timing	Overall risk of bias	Overall applicability
Seifer et al. (40)	Low	Low	Low	Low	Low	High
Seifer et al. (39)	Low	Low	Low	Low	Low	High
Stutz et al. (45)	Low	Low	Low	Low	Low	High
Sloan et al. (42)	Unclear	Low	High	Low	High	High
Sloan et al. (43)	Unclear	High	High	High	High	High
Burwinkel et al. (44)	Unclear	High	Low	Low	High	High
Schroeder et al. (46)	Unclear	High	Low	Unclear	High	High

## 6 Implementation challenges

The clinical evidence reviewed in Sections 4 and 5 was generated under study conditions that differ from how most consumers encounter OTC hearing aids. Moving from clinical trials to everyday use introduces challenges related to professional involvement, device usability, consumer decision-making, and data governance. This section reviews the evidence on these implementation factors.

### 6.1 Professional involvement in the OTC model

Although OTC hearing aids were designed to be used without professional assistance, evidence from multiple sources suggests that most users still seek or benefit from professional involvement. In the MarkeTrak 2025 survey, approximately 80% of OTC hearing aid owners reported receiving some type of professional service, and those who did were more satisfied than those who did not (31). Singh and Dhar (47) surveyed 1,037 US adults aged 50 and older without hearing aid experience and found that 84% would pursue hearing healthcare through an in-person model rather than online. People who were older, had higher income, or were not interested in hearing aids were less likely to pursue online pathways.

Davis et al. (48) surveyed 111 primary care physicians and found that 91.9% viewed OTC hearing aids positively and 90.1% wanted to be involved in hearing healthcare, but 61.3% reported being unfamiliar with OTC hearing aids and confidence in counseling patients was low. Participants answered only 5 out of 10 OTC knowledge questions correctly on average. These findings suggest that if primary care is expected to play a role in OTC hearing aid guidance, targeted training is needed.

Remote support tools may reduce the need for in-person visits but do not eliminate it. Convery et al. (49) compared app-based remote fine-tuning with in-person follow-up in 30 hearing aid users over a six-week field trial and found no significant difference in outcomes between groups. However, nearly half of the remote requests involved problems requiring provider advice or physical device modifications rather than software adjustments, reinforcing the continued need for in-person access. More broadly, remote audiological services have been shown to be viable for hearing screening, diagnostic testing, and rehabilitation, with benefits including improved access to care, increased follow-up rates, and reduced travel time and costs (50). This teleaudiology infrastructure could support OTC hearing aid users who need professional guidance without requiring a clinic visit.

### 6.2 Usability for the target population

OTC hearing aids are intended primarily for older adults, a group that may face challenges with the technology required for self-fitting. Coco et al. (51) assessed OTC hearing aid management skills in 82 older adults and found that 68% were unable to complete all required tasks without error. The most difficult tasks were adjusting volume, pairing with a smartphone, and replacing the wax guard, while new users performed significantly worse than experienced users on putting the hearing aids on. Salwei et al. (52) conducted formative and summative usability testing of an OTC hearing aid for adults with mild to moderate hearing loss, evaluating both safety and usability of the device. Hay-McCutcheon et al. (53) described an approach to increasing OTC access in rural Alabama communities, where access to hearing healthcare is limited.

These usability findings are consistent with the gain analysis data from Knoetze et al. (28) (reviewed in Section 4.2), which showed that OTC users tend not to increase amplification over time and that self-selected gain levels are lower than clinical targets, particularly at higher frequencies. Together, these results suggest that the current self-fitting interfaces may not guide users toward optimal amplification.

For people with cognitive impairment, the challenges are greater. Urbanski et al. (54) explored OTC hearing aid feasibility for community-dwelling older adults with dementia through interviews with 45 participants including people with dementia, caregivers, and geriatric professionals. While participants identified increased accessibility and affordability as facilitators, barriers included difficulty assessing hearing aid candidacy from self-reports, caregiver uncertainty about device programming, and concerns about long-term device management burden.

### 6.3 Consumer perspectives and adoption patterns

Large-scale survey data provide early evidence on OTC adoption patterns. Picou and Huang (55) analyzed MarkeTrak 2025 data from nearly 5,000 adults with perceived hearing difficulties. OTC hearing aids added only 4 percentage points to overall hearing device adoption, a smaller impact than earbuds with hearing support features. Prescription hearing aid adoption rates remained stable at approximately 39%. OTC devices were more affordable, but most OTC owners still reported using some form of professional support.

Jilla and Jorgensen (56) further analyzed MarkeTrak 2025 data on adoption trends and found that OTC devices served as a critical access point for first-time hearing aid users, particularly younger, more diverse, and cost-sensitive populations. Traditional hearing aids remained preferred among individuals with more severe hearing loss and those seeking professional support. Dobyan and Kihm (57) reported that overall hearing device adoption, including earbuds with hearing improvement features, has increased to over half of adults with perceived hearing difficulty, with satisfaction levels remaining stable and financial assistance for hearing aids increasing since the previous survey.

Knoetze et al. (58) explored cost-related perspectives from both prescription and OTC hearing aid users. Both groups identified high cost and lack of insurance coverage as the main barriers to hearing aid uptake. OTC users more often cited affordability as an advantage, while prescription users more often reported external financial support such as insurance or family assistance.

### 6.4 Data privacy

OTC hearing aids that connect to smartphone applications collect personal data including audiometric profiles, usage patterns, and in some cases health sensor readings. When manufacturers sell directly to consumers without involving a healthcare provider, the data they collect may fall outside the scope of health privacy regulations such as HIPAA, which applies to covered entities and their business associates rather than to consumer electronics companies (59). This regulatory gap has been noted in the broader consumer and digital health technology literature, but hearing-specific research on data governance practices is lacking.

## 7 Discussion

The evidence reviewed in this paper shows that OTC hearing aids represent a meaningful but limited step toward addressing the hearing healthcare access gap. Self-fitting produces clinically significant benefit for most users. In most noninferiority trials, self-fit methods produced outcomes equivalent to professional fitting (17, 20, 24). One trial, in which the OTC arm was simulated using prescription devices, found that audiologist fitting achieved significantly better outcomes than OTC use (25), although OTC outcomes were still positive. This pattern suggests that the benefit of professional fitting may depend on the specific population, care pathway, and outcome measure used, rather than being uniformly superior. Evidence for preprogrammed OTC devices remains limited (see Section 4.4). Similarly, early evidence for consumer electronics hearing aids suggests high usability but limited audiological benefit (see Section 4.3).

These positive findings have two limits. Several trials were run with support from the device makers, who supplied the hearing aids. This includes the largest trial to date (20) (*n* = 584), which used the same hearing aids in all groups rather than devices sold over the counter. In addition, most participants took part through clinical studies and may not behave like a person who buys an OTC device with no help from a professional. Together with the other limitations in Section 4.4, this means the trial results may not apply directly to everyday OTC use.

More broadly, the introduction of OTC hearing aids reflects a shift in the values of hearing healthcare. Menon et al. (60) conducted a qualitative content analysis of regulatory and critique documents that shaped the OTC category and found inconsistency between the motivations for creating OTC hearing aids (reducing barriers) and the actual regulatory implementation. This tension between consumer autonomy and professional oversight is visible across the evidence reviewed here, from the finding that most OTC users still seek professional support to the usability challenges discussed in Section 6.

Health-monitoring sensors in hearing aids present a different challenge. Accelerometry have reached technical accuracy comparable to research reference methods for gait and to consumer wearables for energy expenditure (39, 45), but most of this validation has come from the hearing aid manufacturers themselves, such as WS Audiology, Starkey, and Sonova, therefore independent replication is still limited. Moreover, no study has linked hearing aid sensor data to improved patient outcomes (see Section 5). Until that evidence exists, hearing aid sensors remain a technically validated capability without a proven clinical application.

Two emerging research directions connect the OTC and sensor domains. First, recent work combining hearing aid acoustic data with cardiovascular measurements from wristbands has shown that listening environments affect heart rate (61, 62), raising the possibility that hearing aids could contribute to cardiovascular monitoring if ear-based PPG sensors can be validated. Second, hearing aids may have broader health effects beyond hearing improvement. Goodwin et al. (63) used wrist-worn accelerometry to assess whether hearing aid provision changes physical activity levels in older adults, while hearing aid accelerometers themselves have shown promise for step counting and clinical mobility assessment (42). Together, these lines of work suggest that hearing aids could both monitor and potentially improve physical health outcomes, though the evidence for both remains preliminary.

Looking further ahead, these devices could connect into wider care, with sensor-equipped hearing aids feeding into teleaudiology, remote monitoring, and interdisciplinary health data once the foundational evidence is established. This broader integration remains a prospect rather than a present capability, and it will depend on resolving interoperability between proprietary platforms and on clear data governance.

Research priorities identified in the literature include determining which outcome measures are best suited to evaluating OTC hearing aids, clarifying how effective self-fitting algorithms are relative to prescription targets, and identifying which users are most likely to benefit from and successfully navigate the OTC service delivery model (3). The evidence reviewed here further suggests a need for longer-term follow-up, for testing self-fitting across users with differing levels of technology literacy, and for independent trials using commercially available OTC devices rather than research-modified prescription hearing aids. In the sensor domain, the key need is for prospective studies that test whether sensor readings can guide care in a way that improves patient outcomes.

The relationship between hearing intervention and cognitive health is also an important area for future OTC research. The ACHIEVE trial (*n* = 977) found that hearing intervention with prescription hearing aids did not reduce 3-year cognitive decline in the overall cohort, but a prespecified analysis showed a significant benefit in participants at higher risk for cognitive decline (64). A secondary analysis found that hearing intervention slowed decline by 62% among those in the top quartile of predicted risk (65). Whether OTC hearing aids, which reach a different population through a different pathway, can produce similar cognitive benefits is untested but represents a priority for future research.

A cost-effectiveness analysis projected that OTC hearing aids would be cost-effective over a lifetime provided they achieve at least 55% of the quality-of-life benefit of traditional hearing aids (66), suggesting that even modestly effective OTC devices could represent good value at current price points. Chung and Zeng (67) have proposed fitting algorithms that could enable OTC amplification without a formal audiogram and have argued that smartphones and wireless earbuds could functionally serve as OTC hearing aids, potentially expanding access in low- and middle-income countries.

## 8 Conclusion

This review examined the clinical evidence for OTC hearing aids and the validation status of health-monitoring sensors in hearing aids, covering the period from 2020 to 2026. The FDA's 2022 OTC rule created a new access pathway for adults with perceived mild to moderate hearing loss, and early evidence indicates that self-fitted OTC hearing aids provide meaningful benefit, though outcomes may vary depending on the care pathway and population served. Consumer adoption has been incremental rather than transformative, with most OTC users still seeking professional support.

Sensor capabilities in hearing aids have advanced from concept to technical validation, with accelerometry-based gait analysis matching research reference methods and energy expenditure estimation matching consumer wearables. Multi-sensor platforms that measure vital signs from hearing aids have been shown to be technically feasible. However, the gap between sensor validation and clinical benefit remains unresolved, and no study has linked hearing aid sensor data to improved patient outcomes.

The convergence of OTC access and sensor integration means that hearing aids are beginning to function as health monitoring platforms worn throughout the day. If sensor accuracy is confirmed through clinical outcome studies, hearing aids could become relevant to disciplines beyond audiology, including geriatric medicine, cardiovascular monitoring, and fall prevention. This would position hearing care within broader interdisciplinary health teams and expand the clinical value of a device that millions of older adults already wear. Reaching that point will depend on prospective clinical trials linking sensor data to health outcomes, standardized validation protocols, and regulatory frameworks that can accommodate dual-purpose devices. Until that evidence is in place, claims about hearing aids as comprehensive health platforms are not yet supported by outcome data.
