# Supplementary material for: Over-the-counter hearing aids and integrated health-monitoring sensors: a review of clinical evidence and implementation
Source: Front Digit Health. 2026 Jul 29;8:1771022. doi: 10.3389/fdgth.2026.1771022 (PMC13463186; doi:10.3389/fdgth.2026.1771022)
Supplement: Supplementary file 1 [file Datasheet1.pdf]

Supplementary material

This review is a narrative review conducted with a systematic, documented literature search. PRISMA 2020 (1) is used as a reporting framework. Each item below is marked either as "reported", with its location in the manuscript, or as "not applicable" because no meta-analysis or other statistical pooling was performed. Items specific to quantitative synthesis are the ones marked "not applicable".

Supplementary Table 1. PRISMA 2020 checklist

Section	#	Reporting item	Location or status
**Title**	1	Identify the report	Reported (as a review). The title identifies the work as a review, and Section 2 states it is a narrative review. It is deliberately not labeled a systematic review, consistent with its design.
**Abstract**	2	Structured abstract	Reported. Abstract.
**Introduction**	3	Rationale	Reported. Section 1.
	4	Objectives / questions	Reported. Section 1 states the questions addressed across the two areas.
**Methods**	5	Eligibility criteria	Reported. Section 2.2.
	6	Information sources	Reported. Section 2.1 (four databases; date range January 2020 to May 2026; searched May 2026).
	7	Search strategy	Reported. Section 2.1 gives the databases, date range, fields, and the full Boolean search terms for both search domains.
	8	Selection process	Reported. Selection steps are described in Section 2.3. Searches and screening were performed by the first author and independently confirmed by the second author.
	9	Data collection process	Reported. Extracted data items are listed in Section 2.3. Extraction was performed by the first author and confirmed by the second author.
	10a	Outcomes / data items	Reported. Section 2.3 lists the extracted items. No synthesis-specific outcome domains were pre-defined, as no pooling was performed.
	10b	Other variables	Reported. Section 2.3.
	11	Study risk-of-bias methods	Reported. Tools are named in Section 2.5 (RoB 2 for comparative trials, QUADAS-2 for sensor validation studies). Ratings were assigned by the first author and confirmed by the second author.
	12	Effect measures	Not applicable. No quantitative synthesis performed.
	13a	Studies eligible for each synthesis	Not applicable. No synthesis performed.
	13b	Data preparation for synthesis	Not applicable.
	13c	Tabulation / display of individual results	Reported. Individual study characteristics and results are presented in Tables 1 and 2.
	13d	Synthesis methods / meta-analysis	Not applicable. No meta-analysis or statistical synthesis.
	13e	Exploration of heterogeneity	Not applicable.
	13f	Sensitivity analyses	Not applicable.
	14	Reporting bias assessment (in synthesis)	Not applicable. No synthesis.
	15	Certainty assessment (synthesis level)	Not applicable. No synthesis. Per-study risk of bias is reported instead (Section 2.5, Tables 3 and 4).
**Results**	16a	Study selection results / flow diagram	Reported. Section 2.3 and the PRISMA flow diagram (Figure 1).
	16b	Excluded studies with reasons	Reported. Exclusion counts are given in the flow diagram.
	17	Study characteristics	Reported. Tables 1 and 2; Sections 4 and 5.
	18	Risk of bias in studies	Reported. Tables 3 and 4.
	19	Results of individual studies	Reported (narrative). Sections 4 and 5 and Tables 1 and 2. Standardized effect estimates were not computed, as no synthesis was performed.
	20a	Synthesis results, contributing studies	Not applicable. No synthesis.
	20b	Statistical synthesis results	Not applicable.
	20c	Heterogeneity results	Not applicable.
	20d	Sensitivity analysis results	Not applicable.
	21	Reporting biases	Not applicable. No synthesis.
	22	Certainty of evidence	Not applicable. No synthesis.
**Discussion**	23a	Interpretation	Reported. Section 7.
	23b	Limitations of the evidence	Reported. Sections 4.4 and 7.
	23c	Limitations of the review process	Reported. Section 7.
	23d	Implications	Reported. Sections 7 and 8.
**Other**	24a	Registration	The review was not registered, consistent with a narrative review.
	24b	Protocol	No advance protocol was prepared.
	24c	Amendments	Not applicable. No protocol or registration to amend.
	25	Support / funding	Reported. Funding statement.
	26	Competing interests	Reported. Conflict of Interest statement.
	27	Data / code / materials availability	All data are from published sources cited in the article.

Reference

1. Page MJ, McKenzie JE, Bossuyt PM, Boutron I, Hoffmann TC, Mulrow CD, et al. The PRISMA 2020 statement: an updated guideline for reporting systematic reviews. *Br Med J.* (2021) 372:n71. doi: 10.1136/bmj.n71.
